# Allergic Fungal Rhinosinusitis: A Study in a Tertiary Care Hospital in India

**DOI:** 10.1155/2016/7698173

**Published:** 2016-01-24

**Authors:** Ravinder Kaur, S. Lavanya, Nita Khurana, Achal Gulati, Megh S. Dhakad

**Affiliations:** ^1^Department of Microbiology, Maulana Azad Medical College and Associated Hospitals, New Delhi 110002, India; ^2^Department of Microbiology, Lady Hardinge Medical College and Associated Hospitals, New Delhi 110001, India; ^3^Department of Pathology, Maulana Azad Medical College and Associated Hospitals, New Delhi 110002, India; ^4^Department of ENT, Maulana Azad Medical College and Associated Hospitals, New Delhi 110002, India

## Abstract

The study was conducted to study the occurrence and clinical presentation of allergic fungal rhinosinusitis (AFRS), characterize the same, and correlate with the microbiological profile. Clinically suspected cases of fungal rhinosinusitis (FRS) depending upon their clinical presentation, nasal endoscopy, and radiological evidences were included. Relevant clinical samples were collected and subjected to direct microscopy and culture and histopathological examination. 35 patients were diagnosed to have AFRS. The average age was 28.4 years with a range of 18–48 years. Allergic mucin was seen in all the AFRS patients but fungal hyphae were detected in only 20%. 80% of cases were positive for IgE. All the patients had nasal obstruction followed by nasal discharge (62.8%). Polyps were seen in 95% (unilateral (48.57%) and bilateral (45.71%)), deviated nasal septum was seen in 28.57%, and greenish yellow secretion was seen in 17.14%. Direct microscopy and septate hyphae were positive in 71.42% of cases. 91.4% of cases were positive by culture. 5.7% yielded mixed growth of* A. flavus* and* A. niger*. Prompt clinical suspicion with specific signs and symptoms along with timely sampling of the adequate patient specimens and the optimal and timely processing by microscopy and culture and histopathological examination is a must for early diagnosis and management.

## 1. Introduction

Allergic fungal rhinosinusitis (AFRS), a subset of polypoid chronic rhinosinusitis, is characterized by the presence of eosinophilic mucin with fungal hyphae within the sinuses and a type I hypersensitivity to fungi [1]. Allergic fungal sinusitis is seen to range in a wide percentage of patients with chronic rhinosinusitis from 5 to 10% in some studies [2, 3] to a much higher percentage in others [4]. The disease was initially considered to be prevalent only in northern regions of India but is now reported from other parts of the country also [5].

It is believed that fungal allergens elicit immunoglobulin E- (IgE-) mediated allergic and possibly type III (immune complex) mediated mucosal inflammation in the absence of invasion, in an atopic host [6, 7]. Moreover, when the sensitized individuals are exposed to an environment of high fungal content, symptoms of upper and/or lower airway hyperresponsiveness increase significantly [8]. Generalized sinonasal inflammation in combination with viscid allergic mucin effectively obstructs the normal drainage pathway. Fungi persist locally, stimulating locally destructive immune responses. The process then may expand to involve adjacent sinuses and may produce sinus expansion and bony erosion [9, 10].

To diagnose AFRS, Bent III and Kuhn in 1994 [3] proposed five diagnostic criteria: type I hypersensitivity, nasal polyposis, characteristic findings on CT scan, presence of fungi on direct microscopy or culture, and allergic mucin containing fungal elements without tissue invasion. But in 1994, Cody II et al. [11] reported the Mayo Clinic experience and suggested that diagnostic criteria comprise only the presence of allergic mucin and fungal hyphae or a positive fungal culture.

The criteria for diagnosis of AFRS have undergone numerous revisions; however, most authors agree on the following: the presence in patients with chronic rhinosinusitis (confirmed by CT scan) of characteristic “allergic” mucin containing clusters of eosinophils and their byproducts and the presence of noninvasive fungal elements within that mucin, detectable on staining or culture [2–4, 12]. Most experts also require the presence of documented type 1 (immunoglobulin IgE-mediated) hypersensitivity to cultured fungi and nasal polyposis [2, 3, 12].

There are no clear diagnostic criteria to establish the diagnosis of allergic FRS. With the description of newer categories like eosinophilic fungal rhinosinusitis and eosinophilic mucin rhinosinusitis, it has become more difficult to establish criteria for diagnosis. The laboratory findings in the possible AFRS groups are quite variable and are a source of controversy [13]. Hence, the main objective of this prospective study was to study the occurrence and clinical presentation of allergic fungal rhinosinusitis, characterize the same, and correlate with the microbiological profile.

## 2. Material and Methods

### 2.1. Design and Setting

A prospective study was undertaken to study the occurrence and clinical presentation of AFRS, characterize the same, and correlate it with the microbiological profile of suspected FRS patients.

### 2.2. Participants

Clinically suspected FRS patients (*n* = 75) depending upon their clinical presentation, nasal endoscopy, and radiological evidences from wards and OPDs of our hospital were included in this prospective observational study, after obtaining informed consent from the patients. Relevant clinical history, nasal endoscopy findings, and radiological findings were noted.

### 2.3. Collection of Samples

Relevant clinical samples from the FRS suspected patients, namely, allergic mucin, nasal lavage, exudate from the nasal mucosa, tissue biopsy from nasal polyps, sinus mucosa from middle meatus preoperatively under endoscopic guidance and during paranasal surgery, and venous blood, were received in Department of Microbiology and Pathology. Nasal tissue samples were cut into small pieces using sterile scissors and were sent in normal saline and formalin.

### 2.4. Microscopy, Culture, and Identification

A portion of each of the nasal sample was examined using light microscopy after digestion with 10% potassium hydroxide (KOH) and using fluorescent microscopy after digestion with a mixture of KOH and calcofluor white. The remaining portions of the samples were cultured onto Sabouraud's dextrose agar and Sabouraud's dextrose agar with chloramphenicol and gentamicin. They were incubated at 22°C and 37°C for 4 weeks. Fungal isolates were identified by the colony morphology and microscopic morphology (including Riddle's slide culture) observed on lactophenol cotton blue (LPCB) preparations as per standard recommended procedures [14].

### 2.5. Histopathological Examination

Histopathological examination was done in the Pathology Department and the findings of allergic mucin (consisting of degenerating eosinophils, cellular debris, and Charcot Leyden crystals) inflammation and hyphae and calcification and so forth were recorded. Venous blood sample was taken to evaluate the absolute eosinophilic count and serum total IgE levels of the cases. Eosinophilic count higher than 500 cells per mL was considered as serum eosinophilia while IgE levels were considered raised when the counts were >100 U/mL [13, 15].

### 2.6. Statistical Analyses

Statistical analysis was performed by SPSS software (version 17). Continuous variables are presented as mean ± SD, and categorical variables are presented as absolute numbers and percentages. Categorical variables were analysed using the chi-square test or Fisher's exact test as appropriate. Kappa coefficient was also used to find the agreement between HPE, direct microscopy, and culture variables. For all statistical tests, *p* < 0.05 was considered to indicate a significant difference. All tests of statistical significance were two-tailed.

## 3. Results

35 cases out of 75 cases of suspected FRS were diagnosed to have allergic FRS. The average age was 28.4 years with a range of 18–48 years. Male : female sex ratio was noticed to be 1.18 : 1. 82% of patients were from urban area and 94% were found to be educated. Most cases presented to the hospital in autumn, with an average of 2.75 cases/month followed by winter (an average of 1.83 patients). Mean duration of symptoms was 1.64 years.

All AFRS patients were seen to be suffering from nasal obstruction while nasal discharge was seen in 62.8% of cases with statistically significant association being seen. Other statistically significant associated symptoms were smell disturbances (51.42%), sneezing (31.42%), and loss in vision (11.42%) as shown in Table 1.

The associated comorbidities were bronchial asthma in 14.2% of cases followed by tuberculosis and allergic disorders in 11.42% each and hypertension in 5.71% of cases. 20% of cases had a history of previous nasal surgeries. Statistically significant association was seen in allergic disorders, previous nasal surgeries, and hypertension. Anaemia was seen in 6 (17.14%) cases and found to be statistically associated. Peripheral eosinophilia was significantly seen in 9 (25.71%) cases. Serum total IgE levels were found raised in 80% of AFRS cases (>100 IU/mL).

All cases were subjected to computed tomography scans. Heterogenous opacities were seen in a majority of cases. Bilateral-heterogenous opacities were seen in 60% of cases with a statistically significant association. Mucosal thickening was seen in 22.85% of cases. Pressure effects like bone erosion (31.42% of cases) and intracranial or intraorbital extensions (20% of cases) were also seen. Homogenous opacities on unilateral side and calcification were seen in one case each (Table 2).

On nasal endoscopic examination, polyps were seen in almost 95% of cases, being unilateral in 48.57% of cases and bilateral in 45.71% with a statistically significant association in both. Deviated nasal septum was seen in 28.57% of cases and greenish yellow secretions at the opening of sinuses were seen in 17.14% of cases. Hypertrophy of turbinates was also noticed in around 23%, with middle turbinate hypertrophy (11.42%) showing a statistically significant association (Table 2).

**Figure 1:**
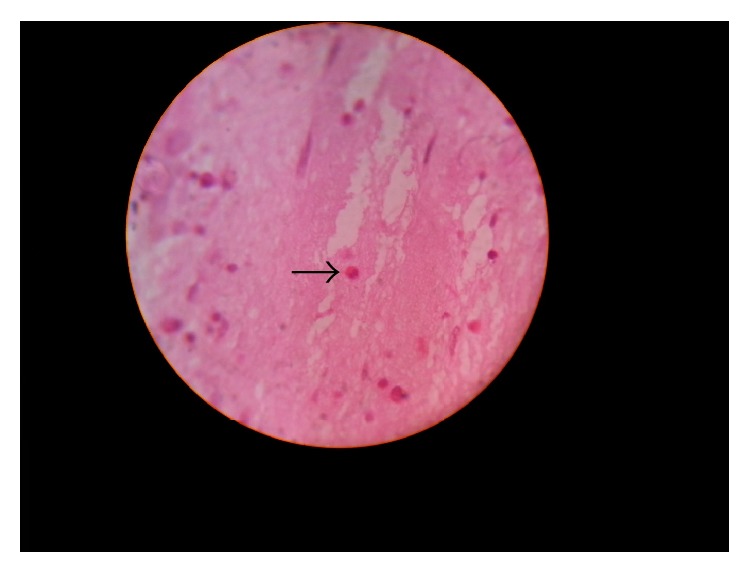
H&E staining of nasal mucin sample showing eosinophils (arrow) at 40x.

All samples sent to the Pathology Department were subjected to histopathological examination using H&E stain (Figure 1) as well as special fungal stains like PAS and Gomori methenamine silver stains. Allergic mucin was seen in all the AFRS cases with statistically significant association. Fungal hyphae were detected in only 7 (20%) cases while acute inflammation and calcification were seen in 1 (2.8%) each.

Direct microscopy was positive in 25 (71.42%) cases and septate hyphae were seen in all these positive cases. 32 out of 35 cases were positive by culture. 2 samples yielded mixed culture, both growing* A. flavus* and* A. niger*. Among cultures,* A. flavus* (27) (77.1%) was the most common species with a statistically significant association followed by* A. niger* (4) (11.4%),* A. fumigatus* (2) (5.7%), and* Bipolaris* species (1) (2.8%) with no statistically significant association being seen.

In 35 cases of AFRS, 7 samples were positive for fungi in both histopathology and culture while in 25 cases, fungi were isolated on culture but no evidence was seen on histopathological examination. Two cases were negative for fungi on culture but were positive by microscopy while 1 sample was negative by both microscopy and culture with slight percentage of agreement being seen between various tests. The percentage of agreement between culture and direct microscopy was 2.5%, between culture and HPE was 4.58%, and between direct microscopy and HPE was 9%.

## 4. Discussions

Allergic fungal rhinosinusitis is a noninvasive form of FRS. Allergic fungal sinusitis is common among adolescents and young adults and is more common in geographical areas of high humidity. Two-thirds of patients are atopic and half suffer from asthma. Two-thirds of allergic fungal sinusitis patients suffer from allergic rhinitis, and approximately 90 percent have increased blood levels of immunoglobulin E (IgE) [16] which was also evident in our study where 80% of AFRS cases have raised serum levels of IgE.

Although there are no unique pathognomonic symptoms, patients often present with unilateral nasal polyposis and thick yellow-green nasal or sinus mucus. The nasal polyposis may form an expansive mass that causes bone necrosis of the thin walls of the sinuses. Should the lamina papyracea of the ethmoid bone be traversed, it may cause proptosis. Polypoid material can also push the nasal septum into the contralateral airway. CT scans often reveal characteristic serpiginous sinus opacification of more than one sinus, mucosal thickening, and erosion of bone, but this does not represent tissue invasion [2, 17]. In addition, allergic fungal sinusitis may be suspected when a patient with nasal polyposis, having no other known disease, responds only to oral corticosteroids.

In our study, 35 cases were diagnosed to have AFRS depending on presence of allergic mucin in histopathology examination and clinical and radiological evidence of allergic fungal rhinosinusitis as well as on the basis of microbiological examination. The mean age of our cases was 28.45 years with a range of 18–48 years, very similar to a study done in Chandigarh in 2002-2003, the mean age being 28 years in their cases of AFRS [13]. However Montone et al. [18] in USA in 2008 found the mean age to be on the higher side, being 45 years with a range of 18–88 years, and M : F ratio was 1.2 : 1 similar to our study.

Many patients with allergic fungal sinusitis have a history of chronic rhinosinusitis and have undergone multiple operations prior to diagnosis [2, 3]. In a study in Brazil in 2002 [19], doctors found a significant association of asthma, previous intolerance to aspirin, and topical corticoid use with AFRS [19]. In our study, previous nasal surgeries were seen in 20% of cases followed by bronchial asthma (14.2%), allergic disorders (11.42%), and hypertension (5.71%). Statistically significant association was seen with allergic disorders, previous nasal surgeries, and hypertension. The recurrent nature of AFRS was demonstrated by Dall'Igna et al. [19], in Brazil where 45.8% of his AFRS cases needed surgical reintervention owing to recurrence of the disease.

Peripheral eosinophilia (>500/mL) was significantly seen in 9 (25.71%) cases and serum total IgE levels were raised in 80% of cases of AFRS tested for the same. Eosinophilia is one of the minor criteria useful for diagnosis of AFRS as defined by Bent III and Kuhn [3]. In a study in USA in 1999 on patients of allergic FRS, elevated total IgE levels were seen in fewer than 33% of AFRS. The possibility of local IgE production in the nasal mucosa might explain the low level of serum IgE in AFRS patients in our study [4].

Heterogenous opacities on bilateral sides were the most common and statistically significant finding in the CT imaging of our AFRS cases (60%) proving the tendency of the disease to be bilateral in nature [20]. Other major CT findings seen in our AFRS cases like near complete opacification of sinuses and hyperattenuating allergic mucin within the lumen of the sinuses seen in the CT of AFRS cases were similar to features described by Aribandi et al. [20], as near complete opacification of sinuses and hyperattenuating allergic mucin within the lumen of the sinuses are seen in the CT of AFRS cases. On nasal endoscopy, polyps were commonly seen and significantly associated with AFRS cases in our study similar to a study done in Brazil in 2002 [19].

To diagnose AFRS, the presence of allergic mucin in histopathologic specimens though not uniformly distributed throughout sinus content is important in addition to the demonstration of fungal elements. However, allergic mucin is not uniformly distributed throughout sinus content. An inadequate sampling may thus pose problems in proper categorization of cases [13]. All the AFRS patients in our study had presence of allergic mucin with statistically significant association consisting of degenerating eosinophils, cellular debris, and Charcot Leyden crystals. Evidence of fungal hyphae by microscopy was only seen in 20% of cases while culture was positive in 91.4% of cases. This might be due to either sparse presence of hyphae or failure of special stains to pick them up, and hence we included these cases of allergic mucin with absence of hyphae in our AFRS cases. Montone et al. [18] in their study in USA in 2008 had observed 74.4% of AFRS patients having histological evidence of fungi and 25.5% of patients for presence of fungi on histopathology being culture positive, adding to the evidence of the presence of fungi in such cases.

In our AFRS cases, hyphae were evident only in 7 cases by histopathology but by culture and microscopy fungi were present in 17 cases. Two cases were positive for fungi by microscopy only and 8 were positive by culture alone. In one case, there was no evidence of fungi but allergic mucin was present with a slight percentage of agreement being seen between various tests in our study. Similar to our study, in a study in Chandigarh in 2007, 57 out of 130 cases of AFRS were positive for fungal elements by both HPE and culture, 15 cases were positive by HPE but negative by culture, 29 cases were negative by HPE but culture positive, and 29 cases were positive neither by HPE nor culture [21].

The reason for presence of fungi in culture in spite of their absence in histopathological examination might be due to the occasionally negative fungal staining when the hyphae were sparse and different areas of tissue sampling, along with the inherent subjectivity and varying expertise involved in many cases as far as microscopy is concerned. The reason for the presence of fungi on histopathological examination but culture negativity might be due to the nonviability of fungi in the samples, different areas of sample collection, and impaction of hyphae in the mucin, thus being unable to come in contact with media [21].

In our study, culture was positive in 32 (91.42%) samples,* Aspergillus flavus* being the most common isolate with a statistically significant association. In many studies in India,* A. flavus *was the most common isolate in AFRS cases [21–23]. A study by Saravanan et al. [13], in Chandigarh, reported that, among the 32 patients in the AFRS group, the most common culture isolate was* A. flavus* (81%), followed by* A. fumigatus* (9%), with* Bipolaris* spp. being isolated in only 2 cases (6%). Meanwhile in the West, in a period (1991–2008), the most common single fungal isolates were* Aspergillus* sp. (34%) with* A. fumigatus*,* A. flavus*, and* A. niger* being most frequent and dematiaceous species (30%) with* Alternaria* spp.,* Bipolaris* spp., and* Curvularia* spp. isolated most often. In cultures with multiple isolates, various combinations of dematiaceous fungi with* Aspergillus* and non-*Aspergillus* spp. have been seen [17]. Our study did not find dematiaceous fungi much among our isolates. This might be due to different geographical distribution of the fungi in different areas depending on local climate temperature and humidity.

It was realised that prompt clinical suspicion in patients of chronic sinusitis with suspicious signs and symptoms along with timely sampling of the adequate patient specimens and the optimal and timely processing of samples by microscopy and culture and histopathological examination will go a long way for early diagnosis and management of these patients.

## Figures and Tables

**Table 1:** Clinical presentations in patients of AFRS (*n* = 35).

Symptoms	Non-AFRS (35)	AFRS (35)	*P* value
	*n* (%)	*n* (%)	
Duration in years (mean)	1.2	1.6	
Nasal obstruction	24 (68.5)	35 (100)	**0.0003**
Headache	19 (54.2)	21 (60)	0.806
Nasal discharge	13 (31.1)	22 (62.8)	**0.05**
Smell disturbances	3 (8.6)	18 (51.4)	**<0.0001**
Loss of vision	11 (31.4)	4 (11.4)	**0.03**
Sneezing	1 (2.8)	11 (31.4)	**0.002**
Proptosis	4 (11.4)	7 (20)	0.51
Fever	8 (22.8)	0	—
Postnasal drip	1 (2.8)	6 (17.1)	0.11
Facial swelling	6 (17.1)	0	—
CNS symptoms	6 (17.1)	0	—
Diplopia	2 (5.7)	4 (11.4)	0.67
Epistaxis	0	3 (8.6)	0.23
Facial pain	2 (5.7)	0	—
Ocular/nasal itching	0	1 (2.8)	0.99

**Table 2:** Diagnostic profile in AFRS cases (*n* = 35).

Findings	Non-AFRS (35)	AFRS (35)	*P* value
	*n* (%)	*n* (%)	
*Computed tomography findings*			
Heterogenous opacities, unilateral	8 (22.8)	13 (37.1)	0.29
Heterogenous opacities, bilateral	8 (22.8)	21 (60)	**0.003**
Homogenous opacities, unilateral	2 (5.7)	1 (2.8)	0.99
Homogenous opacities, bilateral	1 (2.8)	0	—
Mucosal thickening	16 (45.7)	8 (22.8)	0.07
Bone erosion	17 (48.5)	11 (31.4)	0.22
Intracranial/intraorbital extension	15 (42.8)	7 (20)	0.07
Calcification	5 (14.2)	1 (2.8)	0.19
*Nasal endoscopic examination*			
Polyp, unilateral	8 (22.8)	17 (48.5)	**0.04**
Polyp, bilateral	6 (17.1)	16 (45.7)	**0.02**
Deviated nasal septum	4 (11.4)	10 (28.5)	0.13
Secretions, greenish yellow	4 (11.4)	6 (17.1)	0.73
Inferior turbinate hypertrophy	4 (11.4)	4 (11.4)	1
Middle turbinate hypertrophy	0	4 (11.4)	0.11
*Histopathological findings*			
Acute inflammation	6 (17.1)	1 (2.8)	0.10
Allergic mucin	3 (8.6)	35 (100)	**<0.0001**
Fungal hyphae, septate	3 (8.6)	7 (20)	0.30
Calcification	1 (2.8)	1 (2.8)	1
